# Transition From Interferon‐Based Therapy to Direct‐Acting Antivirals in Albanian Children With Chronic Hepatitis C: A 17‐Year Real‐World Experience

**DOI:** 10.1155/av/2405090

**Published:** 2026-10-21

**Authors:** Virtut Velmishi, Ermira Dervishi, Gladiola Hoxha, Blerina Saraci, Donjeta Bali, Mirela Xhafa, Mirzana Kapllanaj, Enkeleda Duka, Lila Shundi, Paskal Cullufi

**Affiliations:** ^1^ Pediatric Department “Mother Teresa” Hospital Tirana Albania; ^2^ Institute of Public Health Tirana Albania cam.ac.uk

**Keywords:** chronic hepatitis C, direct-acting antivirals, interferon, pediatric hepatitis C, ribavirin, sustained virologic response

## Abstract

**Background:**

Direct‐acting antivirals (DAAs) have transformed the management of chronic hepatitis C virus (HCV) infection, including in children. However, real‐world data from resource‐limited settings remain scarce.

**Methods:**

We conducted a retrospective study of children with chronic HCV infection treated at the national pediatric hepatology center in Tirana, Albania, between 2007 and 2024. Patients were grouped according to treatment era: interferon‐based therapy or DAA regimens. Sustained virologic response (SVR), adverse events, and treatment duration were assessed.

**Results:**

Twenty‐nine children were included (17 interferon era; 12 DAA era). SVR was achieved in 83% of patients receiving interferon‐based therapy and in all patients treated with DAAs. Adverse events were more frequent and clinically significant in the interferon group, whereas only mild events were observed in the DAA group. Treatment duration was substantially shorter with DAAs (8–12 weeks vs. 48 weeks).

**Conclusions:**

This real‐world experience reflects the transition toward shorter, better‐tolerated, and highly effective therapies for pediatric HCV infection. While cross‐era comparisons are limited, the findings are consistent with the established efficacy of DAA regimens.

## 1. Introduction

Hepatitis C virus (HCV) infection is a significant global health problem and a major cause of chronic liver disease, contributing substantially to morbidity and mortality worldwide [1]. The overall prevalence of HCV infection in children has been estimated at 0.87%, ranging from 0.34% in Europe to 3.02% in Africa [2]. Vertical transmission represents the most common route of HCV infection in children; the highest risk is observed in infants born to mothers with high HCV viral loads, and this risk is further increased in cases of maternal HIV–HCV coinfection [3]. Between 25% and 40% of infected children spontaneously clear the infection within the first four years of life [4]. Chronic HCV infection is usually asymptomatic during childhood and follows a more indolent course compared with adulthood. The development of cirrhosis and end‐stage liver disease due to HCV infection is rare in children (1%–2%) [5, 6]. However, chronic HCV infection can lead to severe complications in early adulthood, including cirrhosis, hepatocellular carcinoma, and liver failure [6], with a median interval of 33 years after infection. Disease outcomes have been shown to be more favorable in patients treated before the development of advanced liver disease [7].

By December 2008, the combination of pegylated interferon (PEG‐IFN) and ribavirin had been approved by the US Food and Drug Administration (FDA) for use in children older than 3 years. This approval followed the progressive accumulation of pediatric experience, in parallel with adult studies, with interferon‐α (IFN‐α) monotherapy [8–12], combination therapy with IFN‐α and ribavirin [13, 14], and PEG‐IFN monotherapy [15].

The introduction of direct‐acting antivirals (DAAs) has revolutionized the treatment landscape of chronic hepatitis C, particularly in the pediatric population. In 2017, the first DAA‐based regimens sofosbuvir/ribavirin[16, 17] (SOF + RBV) and sofosbuvir/ledipasvir [18–20] (SOF/LDV) were approved by regulatory authorities and subsequently recommended by the World Health Organization and major professional societies for use in adolescents (> 12 years of age). This was followed by the approval of glecaprevir/pibrentasvir (GLE/PIB) [21, 22] and sofosbuvir/velpatasvir (SOF/VEL) [23] in 2019–2020, and by sofosbuvir/velpatasvir/voxilaprevir (SOF/VEL/VOX) and elbasvir/grazoprevir (EBR/GZR) in 2021 [24]. The first DAA regimens approved for children as young as 3 years of age were the fixed‐dose combinations of SOF/LDV [20] and SOF + RBV [17] in 2019, followed by SOF/VEL and GLE/PIB [20] in 2021.

Based on available information online, SOF/LDV (e.g., Harvoni) primarily targets hepatitis C (HCV) genotypes 1, 4, 5, and 6, while GLE/PIB (e.g., Mavyret) is pan‐genotypic as it covers all 6 main HCV genotypes. The regimen SOF/LDV combines sofosbuvir, a nucleotide analog polymerase inhibitor that blocks viral replication, with ledipasvir, an NS5A inhibitor that prevents the virus from assembling, while the GLE/PIB regimen combines glecaprevir, an NS3/4A protease inhibitor that blocks the virus from dividing, with pibrentasvir, an NS5A inhibitor [25].

Despite these advances, real‐world pediatric data from low‐resource settings remain limited. This study describes a single‐center experience over 17 years, capturing the transition from interferon‐based therapy to DAA regimens in Albanian children with chronic HCV infection.

## 2. Materials and Methods

This is a retrospective comparative study that analyzed medical records of children diagnosed with chronic hepatitis C who were treated in our center between 2007 and 2024. The first group, comprising children aged 3 to 15 years (14 boys and 3 girls, mean age 8.2 years), was treated with traditional interferon/ribavirin (IFN/Rib) or PEG‐IFN/Rib for 48 weeks, with SVR assessed 24 weeks after treatment completion (Table 1; Figure 1A). The second group, comprising children aged 4–18 years (10 boys and 2 girls, mean age 12.5 years), was treated with DAAs: half received (ledipasvir/sofosbuvir) for 12 weeks, and the other half received GLE/PIB for 8 weeks (Table 2; Figure 1A).

**Table 1:** The first group of patients treated with Inf/PegInf + Ribavirin.

Patients	Age/sex	Comorbidities	HCV‐RNA^1^ (copies/mL)	Liver biopsy	Treatment	Side effects	HCV‐RNA^2^	Genotype
1	11Y/M	Non‐Hodgkin lymphoma	1.5 × 10^6^	A1F1^3^	Ifn α2b/Ribavirin	Leucopenia	Not detected	—
2	5Y/M	Non‐Hodgin lymphoma	1.8 × 10^6^	A2F2	Ifn α2b/Ribavirin	None	Detected	1b
3	5Y/M	Testicular teratocarcinoma	761000	A2F0	Ifn α2b/Ribavirin	None	Not detected	—
4	6Y/F	Medulloblastoma	6.3 × 10^4^	A1F2	Ifn α2b/Ribavirin	Leucopenia,headache	Not detected	—
5	12Y/M	Testicular seminoma	9.2 × 10^4^	A2F1	Ifn α2b/Ribavirin	Fever	Not detected	—
6	11Y/M	Leukemia	3600	A1F1	Ifn α2b/Ribavirin	None	Not detected	—
7	10Y/F	Non‐Hodgkin lymphoma	1.1 × 10^6^	A3F2	Ifn α2b/Ribavirin	None	Not detected	1b
8	6Y/F	Leukemia	1.8 × 10^6^	A2F1	Ifn α2b/Ribavirin	None	Not detected	—
9	3Y/M	Hemolytic anemia	1.5 × 10^6^	A1F2	Ifn α2b/Ribavirin	None	Not detected	—
10	8Y/M	Gaucher	6.8 × 10^5^	A2F1	Ifn α2b/Ribavirin	None	Detected	—
11	8Y/M	Leukemia	1.6 × 10^6^	A1F1	Ifn α2b/Ribavirin	Myalgia	Not detected	1b
12	11Y/M	Unknown	1.8 × 10^6^	A2F1	Ifn α2b/Ribavirin	Headache	Not detected	—
13	10Y/M	Leukemia	56700	A2F2	Ifn α2b/Ribavirin	Fever	Not detected	—
14	15Y/F	Pharyngeal Ewing Sarcoma	9.3 × 10^5^	A1F0	Ifn α2b/Ribavirin	None	Not detected	—
15	10Y/M	Leukemia	1.8 × 10^6^	A2F1	PEGIfn α2b/Ribavirin	None	Detected	—
16	10Y/M	Unknown	1.5 × 10^6^	A2F1	Ifn α2b/Ribavirin	None	Not detected	—
17	8Y/M	Cystic fibrosis	2.5 × 10^6^	A2F2	PEGIfn α2b/Ribavirin	Fever, cramps, vomiting	Not detected	—

^1^HCV‐RNA at the beginning of treatment.

^2^HCV‐RNA at 24 weeks after completion of treatment.

^3^METAVIR is a histopathological score which expresses the rank of inflammation (A) and hepatic fibrosis (F) in cases of hepatitis C, etc. Missing ‐A0, A1‐minimum, A2‐moderate; A3‐severe; F0‐no fibrosis; F1‐portal fibrosis non‐septal l; F2‐portal fibrosis with few septal fibrosis; F3‐ septal fibrosis without cirrhosis, F4‐cirrhosis.

**FIGURE 1:**
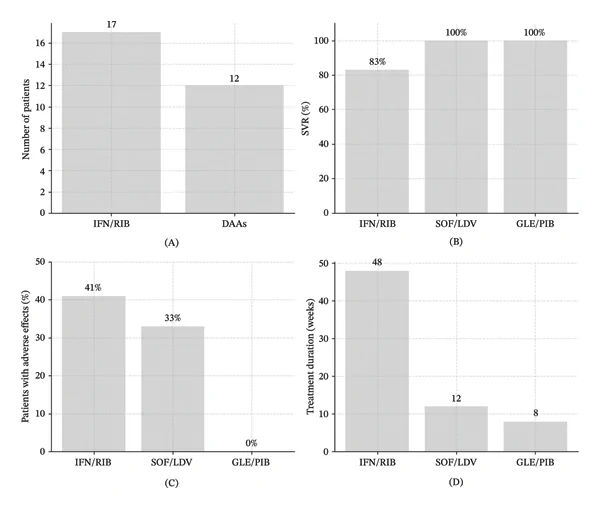
(A) Study group size; (B) treatment efficacy; (C) adverse effects; and (D) duration of treatment.

**Table 2:** The second group of patients treated with DAA.

Patients	Age/sex	Weight	Previous treatment	Comorbidities	HCV‐RNA (IU/mL)	Fibroscan	DAA	Treatment duration	Side effects	SVR 12	Genotype
1	10Y/M	41kg	PIfn + Rib	—	9,246,965	F1	SOF/LDV	12 Weeks	—	ND^∗^	1
2	11Y/M	45kg	—	Neuroblastoma	687,818	F1	SOF/LDV	12 Weeks	—	ND	1b
3	14Y/M	46kg	PIfn + Rib	Leukemia	618,316	F2	SOF/LDV	12 Weeks	Headache	ND	1b
4	15Y/M	55kg	—	Leukemia	116,000	F1	GLE/PIB	8 Weeks	—	ND	1b
5	12Y/M	39kg	—	Hemolytic anemia	80,401	F1	SOF/LED	12 Weeks	Headache, Abd pain	ND	1b
6	16Y/M	62kg	PIfn + Rib	Burkitt lymphoma	27,484	F0‐1	SOF/LED	12 Weeks	—	ND	1b
7	15Y/M	83kg	—	Leukemia	116,573	F1	GLE/PIB	8 Weeks	—	ND	1b
8	4Y/F	15kg	—	—	1405	F0‐1	GLE/PIB	8 Weeks	—		1b
9	18Y/M	94kg	Ifn + Rib	—	1,400,295	F2	GLE/PIB	8 Weeks	—	ND	1b
10	9Y/M	25kg	—	Cystic fibrosis	449,680	F1	GLE/PIB	8 Weeks	—	ND	1b
11	13Y/M	38kg	—	—	184,373	F0‐1	SOF/LDV	12 Weeks	—	ND	1b
12	14Y/M	60kg	—	Hypothyroidism	7,440,000	F1	GLE/PIB	8 Weeks	—	ND	1b

Abbreviation: ND, Not Detected.

The patients in the first group are selected from November 2007 to March 2010 at the Pediatric Gastrohepatology Service, Mother Teresa Hospital, Tirana, Albania. We have treated 17 children diagnosed with chronic hepatitis C [26]. The diagnosis of these children is established through the anti‐HCV testing, confirmed later with HCV‐RNA. We have not had any case of HCV associated with hepatitis B, D, HIV, or other pathology, which would severely damage liver function. The majority of the patients, 14 in total, came from the oncohematology service where they were treated for their illnesses. All these children were in full remission of their basic disease or were considered cured and had at least 12 months without treatment with cytostatics or immunosuppressive agents. All the patients who came from oncohematology service have received multitransfusions. Two cases showed hepatitis C with an unknown transmission route, and a child was diagnosed with Gaucher disease treated with enzyme replacement therapy. No cases of vertical transmission were identified. Treatment was administered according to a protocol combining interferon alfa‐2b (two children received PEG‐IFN alfa‐2b) and ribavirin. Interferon alfa‐2b was administered at a dose of 3 MIU/m^2^ subcutaneously three times a week, while PEG‐IFN alfa‐2b was administered at a dose of 1.5 mcg/kg subcutaneously once weekly. Ribavirin was administered at a dose of 15 mg/kg/day divided into two doses (Table 1).

The patients in the second group were selected between May 2022 and December 2024. Four of those patients failed the standard treatment with IFN/PEG‐IFN and ribavirin. The second group, comprising children aged 4–18 years (10 boys and 2 girls), was treated with DAAs: half received (ledipasvir/sofosbuvir) for 12 weeks, and the other half received GLE/PIB for 8 weeks. We compared the following: (1) sustained viral response (SVR): SVR24 in the first group versus SVR12 in the second group; (2) adverse events: comparison of side effects between patients treated with IFN/Rib and those treated with DAAs; and (3) treatment duration: evaluation of the length of therapy between the two treatment regimens.

### 2.1. Statistical analysis

Continuous variables were expressed as mean ± SD or median (IQR), as appropriate. Comparisons were performed using the Mann–Whitney *U* test for continuous variables and Fisher’s exact test for categorical variables. A *p* value < 0.05 was considered statistically significant.

### 2.2. Ethics

The study was conducted in accordance with the Declaration of Helsinki and was approved by the local ethics committee.

## 3. Results and Discussion

This study covered a relatively long period of 17 years (2007–2024). Since there is only one national center in our country where children with chronic HCV are treated, it was straightforward to identify patients who had been treated with the conventional therapy of the time (IFN/PEG‐IFN + ribavirin) and, more recently, patients treated with DAAs. In the first group treated with traditional therapy, we considered SVR24 as the gold standard, whereas in the second group, we evaluated SVR12. Baseline HCV‐RNA levels were comparable between the two treatment groups. The median baseline viral load in the interferon‐based cohort was 5.74 log10 IU/mL (IQR 5.46–6.34), while in the DAA‐based cohort, it was 5.85 log10 IU/mL (IQR 5.26–6.64), with no significant difference (*p* = 0.78). SVR was achieved in 14 of 17 patients (83%) treated with interferon‐based regimens and in all 12 patients (100%) treated with DAAs (Figure 2). The superiority of DAA therapy was in fact expected, as numerous studies have demonstrated this [27–30].

**FIGURE 2:**
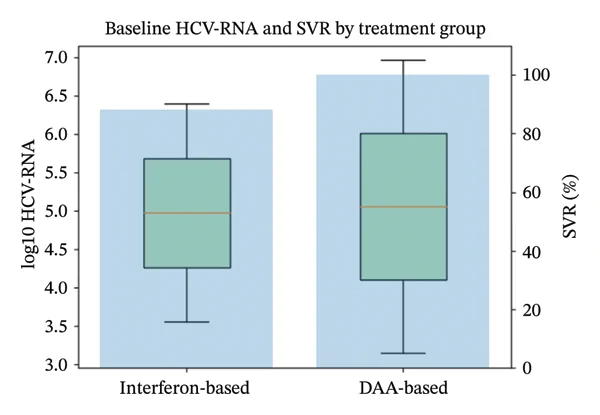
Baseline HCV‐RNA levels showed a wide but overlapping distribution between the interferon‐based and DAA‐based treatment groups, with no statistically significant difference observed (*p* > 0.78).

We had the opportunity to use two types of DAA regimens, and their selection was random. The first patients in the second group started therapy with SOF/LDV, as it was among the first regimens approved for children over 3 years of age. The other regimen was GLE/PIB, which is pangenotypic, unlike SOF/LDV (Table 2). In addition to being pangenotypic, GLE/PIB had another advantage, namely, a shorter treatment duration of 8 weeks compared with 12 weeks for SOF/LDV and, naturally, much shorter than the 48‐week course used in the first group with PEG‐IFN and ribavirin (Figure 1D). Regarding virological response, we achieved SVR12 = 100% with both DAA regimens and SVR24 = 83% in the first group treated with PEG‐IFN plus ribavirin (Figure 1B). A 2018 study on SOF/LDV reported an SVR12 of 99% in 92 children aged 6–12 years, where 70% of patients experienced side effects such as headache, fever, and abdominal pain [28]. Another study from 2020, including 47 adolescents treated with GLE/PIB, reported SVR12 = 100%, but approximately 70% of patients also developed adverse effects such as nasopharyngitis, upper respiratory tract infections, and headache [28].

Regarding adverse events in our cohorts, it is clear that in the DAA group they were milder and less frequent. In both groups, we did not have any cases requiring treatment discontinuation; however, in the IFN/PEG‐IFN plus ribavirin group, more evident adverse effects were observed in 41% of cases, such as leukopenia, headache, and myalgia (Table 1). In contrast, in the DAA group, adverse effects were milder, such as headache or abdominal pain in 33% of patients treated with SOF/LDV [18, 19], whereas therapy with GLE/PIB was very well tolerated and without adverse effects (Figure 1C). Headache, fatigue, and nausea are the most commonly reported side effects of DAA therapy, with no substantial differences between regimens, as shown in several pediatric studies and supported by larger studies in adults [19–22].

Vertically transmitted HIV/HCV coinfection has been associated with a lower likelihood of spontaneous HCV clearance and a higher risk of progressive liver disease compared with HCV monoinfection [31–36]. Reactivation of HBV infection has been reported in some adult studies during DAA treatment; therefore, close monitoring of patients with HCV/HBV coinfection is recommended. Successful use of DAAs has also been reported in pediatric transplant recipients (liver or kidney transplantation) [34–36]. In both of our study groups, we had no cases with coinfection or organ transplantation.

Since genotype 1b predominates in our setting, we indirectly assume that this genotype was also prevalent in the first group, despite the fact that at that time genotyping could be performed in only a limited number of patients due to laboratory constraints and cost. In neighboring Italy, genotype 1a historically predominated; however, recent reports indicate a decline in its prevalence and an increase in genotypes 3 and 4 [37, 38]. In our cohort, especially in the second group, genotype 1b was clearly predominant (Table 2).

FibroScan is an excellent noninvasive method that monitors the degree of fibrosis and its regression after therapy; however, evidence regarding precise cutoff values in the pediatric population remains limited [39–41]. Some studies, mainly in adults, have demonstrated significant correlations between FibroScan values and liver function tests such as AST [42–44]. Hepatic fibrosis generally progresses slowly, but cases of cirrhosis in children have been reported in 1%–2% before the age of 20 years, and if left untreated, the risk of advanced fibrosis increases in adulthood.

Regarding treatment duration, it is evident that DAA therapy has a significantly shorter duration compared with IFN or PEG‐IFN plus ribavirin (Figure 1D): 8 weeks for GLE/PIB, 12 weeks for SOF/LDV, and 48 weeks for IFN/PEG‐IFN + ribavirin. In addition to the shorter duration, another advantage of DAAs is oral administration, compared with the subcutaneous administration of IFN or PEG‐IFN. In a small number of cases, capsule swallowing remains a challenge in younger children. Some recommendations suggest that in such cases the granules inside the capsule may be administered with suitable food and should not be chewed due to their bitter taste [45, 46]. In our DAA group, the youngest child was 4 years old and weighed 15 kg; she was able to swallow the weight‐appropriate GLE/PIB capsule daily for 8 weeks, achieving excellent outcome.

## 4. Limitations

This study has several limitations. The small sample size limits statistical power and generalizability. The retrospective design introduces potential selection bias. The two groups represent different treatment eras, leading to temporal bias and limiting direct comparability. Genotyping was not available for all patients in the interferon group.

## 5. Conclusion

In this real‐world cohort, treatment with DAAs was associated with high cure rates, shorter treatment duration, and improved tolerability. Although comparisons with interferon‐based therapy are limited by study design, these findings reflect the substantial progress in pediatric HCV management and support current recommendations favoring DAA therapy.

## Author Contributions

Virtut Velmishi drafted the manuscript. Ermira Dervishi and Gladiola Hoxha performed the statistical analysis. Donjeta Bali, Mirela Xhafa, Enkeleda Duka, and Mirzana Kapllanaj contributed with cases from oncohematology service. Blerina Saraci interpreted FibroScan images. Lila Shundi was involved with HCV‐RNA testing and genotyping analyses. Paskal Cullufi helped to draft the manuscript.

## Funding

No funding was received for this manuscript.

## Disclosure

All authors read and approved the manuscript.

## Conflicts of Interest

The authors declare no conflicts of interest.

## Data Availability

The data that support the findings of this study are available from the corresponding author upon reasonable request.
